# Development and Elemental Analysis of a Novel Strontium-Doped Nano-Hydroxyapatite Paste and Evaluation of Its Remineralization Potential: An In Vitro Study

**DOI:** 10.7759/cureus.48650

**Published:** 2023-11-11

**Authors:** Ratheesh Rajendran, Delphine P Antony

**Affiliations:** 1 Conservative Dentistry and Endodontics, Saveetha Dental College, Saveetha Institute of Medical and Technical Sciences, Saveetha University, Chennai, IND

**Keywords:** strontium-doped nano-hydroxyapatite, caries detection, elemental analysis, sem-edax, remineralization

## Abstract

Background

Early detection and non-invasive methods to treat early caries lesions using new remineralizers are at the heart of today’s caries management. The goal of the study is to develop a novel strontium-doped nano-hydroxyapatite paste, analyze its chemical composition, and evaluate the remineralization potential.

Methodology

Co-precipitation was used to create strontium-doped nano-hydroxyapatite, which was used to make dentifrice. Scanning electron microscopy (SEM) and energy dispersive X-ray analysis (EDAX) were used to analyze the elements in the recently produced strontium-doped nano-hydroxyapatite. From the extracted tooth’s base surface, 30 enamel samples measuring 4 x 4 x 1 mm were created. The mean calcium/phosphorus of all healthy samples and after demineralization were assessed using SEM-EDAX analysis. A new blend of strontium-doped nano-hydroxyapatite was used to remineralize the samples after which the average calcium and phosphorus content was determined.

Results

When compared to the mean calcium and phosphorus values of the demineralized specimen, the mean calcium and phosphorus values after remineralization using strontium-doped nano-hydroxyapatite were greater and statistically significant (p = 0.001). Using the one-way analysis of variance and Tukey’s post hoc test, the mean calcium and phosphorus values of the sound enamel specimen, demineralized specimen, and remineralized specimen were compared.

Conclusions

The novel strontium-doped hydroxyapatite paste showed good remineralization potential. SEM-EDAX evaluation showed favorable topographic changes in enamel.

## Introduction

Dental caries is one of the major public health issues, and efforts to lower patient risk of caries are still being made. Early caries detection as well as noninvasive approaches to the management of early lesions with new remineralizers are at the heart of caries management. Various techniques for applying remineralizers to early lesions are just beginning to aim at controlling demineralization, thereby promoting remineralization. Although there are many remineralization techniques, the use of fluoride is one of the main application systems. Gels, varnishes, and fluoride-releasing materials have been used to inhibit demineralization and induce remineralization of high-risk dental areas with oral hygiene guidelines and regimen control eating [1-3]. The lack of available calcium and phosphate ions limits the use of fluoride therapy for remineralization [4-6].

To release elements that promote enamel and dentin remineralization, bioactive compounds based on the milk protein casein-phosphopeptide (CPP) with amorphous calcium phosphate (ACP) have been created. High quantities of calcium and phosphate ions have been shown to preserve the supersaturation of enamel minerals, promoting the remineralization of early lesions [7]. Recently, hydroxyapatite nanoparticles have been applied for remineralization. Due to its homogeneous particle size, nano-hydroxyapatite (nHAp) releases Ca^2+^ ions at a faster rate and has better functional characteristics [8]. nHAp is a better alternative to fluoride that has no negative side effects. In contrast to fluoride, which is known to produce supermineralization of the surface layers and does not strengthen teeth from the inside, nHAp has the ability to induce interior tooth mineralization, which can be further enhanced by natural saliva therapy [9].

Due to its success in treating tooth sensitivity, remineralization, and enamel degradation, nHAp has been promoted as a toothpaste. However, the main pitfalls of nHAp are its fragility, high crystallinity, low solubility at neutral pH, and requirement for acidic pH to dissolve [10]. It has been observed that a wide variety of ions, including cations and anions, can replace the calcium (Ca), phosphate, or hydroxyl ions in the structure of the produced HAp [11]. The degree of crystallinity and some material properties, notably phase stability and reactivity, vary when substitutes, such as strontium-substituted hydroxyapatite (more than 10 mol% substitution) and carbonated hydroxyapatite (greater than 10 mol% substitution), are added. These substitutes are more soluble than nHAp [12].

The result inferred by Krishnan et al. revealed strontium-doped nHAp as a solution that is superior to CPP-ACP cream and nHAp toothpaste in surface repair over demineralized enamel [12]. However, preparing a denser material such as toothpaste while incorporating this granule into its formulation would be beneficial due to its greater ability to stay on the tooth surface and ease of application similar to regular toothpaste.

The purpose of our study was to incorporate strontium-doped nHAp into a toothpaste form that provides more retention on application, to analyze its chemical composition, and to evaluate the remineralization potential of the novel strontium-doped nHAp toothpaste.

## Materials and methods

Preparation of strontium-doped nanohydroxyapatite

By co-precipitation, strontium-doped hydroxyapatite was created. A 50 mL solution with a 1:1 ratio of calcium nitrate tetrahydrate (0.57 M) and strontium nitrate (0.18 M) was made. For 15 minutes, the solution was swirled. A 50 mL dropwise addition of ammonium dihydrogen orthophosphate (0.45 M) aqueous solution was done after stirring. By introducing ammonia at 80°C, the pH was maintained at 10. For 24 hours, the reaction was allowed to continue while being stirred. The resultant suspension underwent centrifugation, lyophilization, and washing with distilled water. Strontium-doped hydroxyapatite was produced as a result.

Preparation of the paste

The dentifrice was made up of the following ingredients in the following proportions: 15% deionized water, 30% sorbitol and glycerine as a humectant, 2% sodium lauryl sulfate as detergent, 3% sodium alginate as a binder, and 50% strontium-doped nHAp [13].

Tooth samples

A total of 30 intact premolars were collected from healthy young adult patients undergoing orthodontic extractions after obtaining written consent. The study approval was obtained from the Institutional Ethical Committee.

Preparation of enamel specimens

The samples were debrided and soft tissues were placed in normal saline for storage. All the teeth were sectioned using a diamond disc using a slow-speed straight handpiece at 15,000 rpm at the cementoenamel junction to separate the crown portion. The crown’s apical and occlusal surfaces were then removed with the same method. From watch faces, 30 4 × 4 × 1 mm enamel samples were created. Subsequently, the samples were stored in synthetic saliva.

Demineralization of the samples

With McInne’s demineralization solution, which contains 1 mL of 36% hydrochloric acid, 1 mL of 30% hydrogen peroxide, and 0.2 mL of freshly mixed aesthetic ether, samples were demineralized. Before each application, mix 5:5:1 in a dampened dish. All samples were exposed to two demineralization cycles lasting five minutes each, followed by two days of storage in synthetic saliva of pH -6.8.

Remineralization of the samples

After demineralization, samples underwent a remineralization treatment using a brand-new, lab-created toothpaste made with strontium-doped nHAp. For 28 days, every specimen was brushed twice a day for three minutes (at a 12-hour interval) using a motorized toothbrush (Oral B). The samples were cleaned with water after each application of a remineralizing agent before being placed in fake saliva.

Statistical analysis

The data was examined using SPSS for Windows version 22 (IBM Corp., Armonk, NY, USA). The mean values of calcium and phosphorus on the sound enamel specimen, demineralized specimen, and remineralized specimen were compared using the one-way analysis of variance and Tukey’s post hoc test (p < 0.05).

## Results

**Figure 1:**
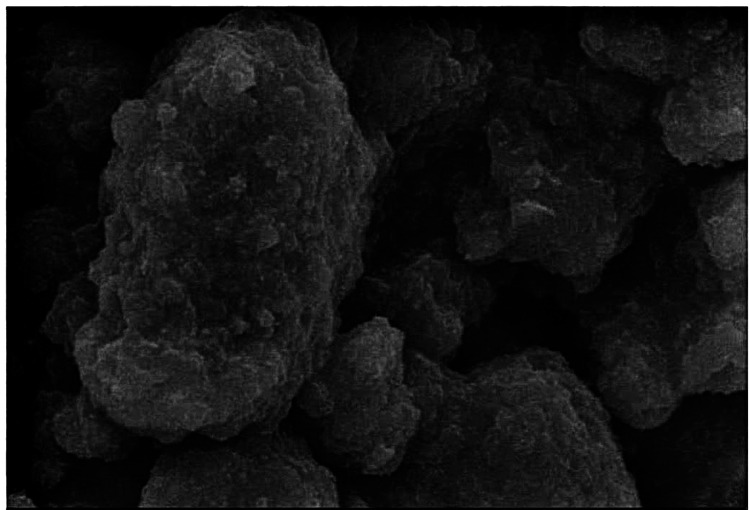
Scanning electron microscope image of strontium-doped nanohydroxyapatite.

**Figure 2:**
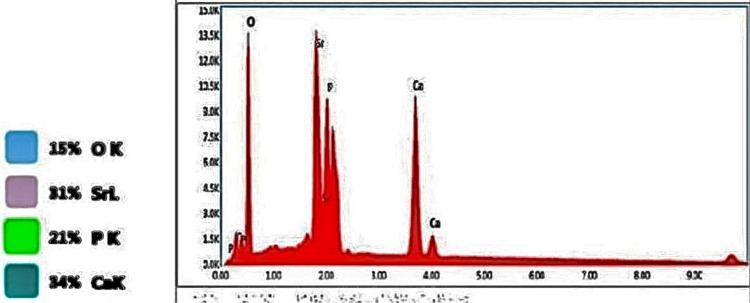
Energy dispersive X-ray analysis of strontium-doped nanohydroxyapatite.

**Figure 3:**
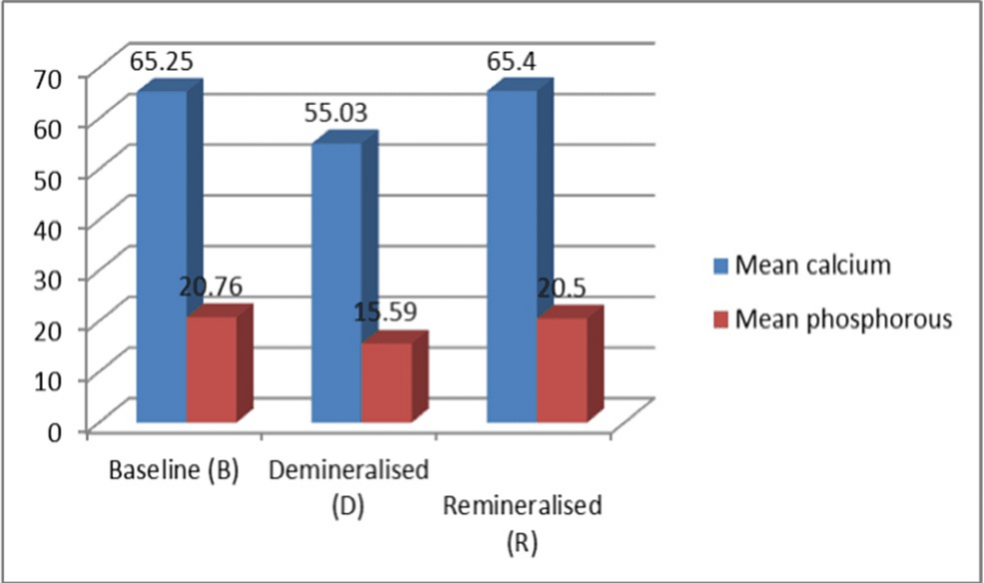
Mean calcium and phosphorus content of sound, demineralized, and remineralized enamel specimens.

**Figure 4:**
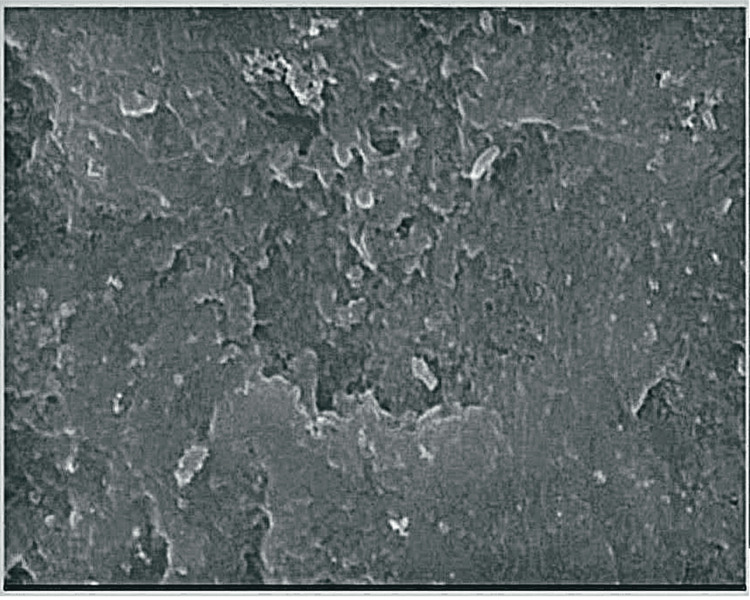
Sound enamel specimen.

The laboratory-synthesized novel strontium-doped nHAp paste was subjected to scanning electron microscopy (SEM) and energy-dispersive X-ray analysis (EDAX) for elemental analysis (Figures 1, 2) SEM was used to assess the surface topography of healthy enamel samples (baseline) and EDAX was used to assess the average calcium and phosphorus levels for healthy tooth enamel (Figures 3, 4, Table 1).

**Table 1:** Comparison of mean calcium and phosphorous of strontium-doped hydroxyapatite was statistically significant (p < 0.05), as calculated by one-way analysis of variance.

Groups	Mean calcium	Mean phosphorous
Baseline (b)	65.25 ± 0.59	20.76 ± 0.81
Demineralized (d)	55.03 ± 0.59	15.59 ± 0.48
Remineralized (r)	65.4 ± 0.86	20.5 ± 0.76
P-value	0.001	0.001

Strontium-doped EDAX paste underwent an elemental analysis, which identified 21.99% of strontium, 25.44% of calcium, and 15.74% of phosphorus (Table 2).

**Table 2:** Elemental analysis of strontium-doped nano-hydroxyapatite paste (energy dispersive X-ray analysis).

Element	Weight %	Atomic %	Error %
O K	36.83	62.29	9.77
SrL	21.99	6.79	1.99
P K	15.74	13.75	4.27
CaK	25.44	17.17	2.78

**Figure 5:**
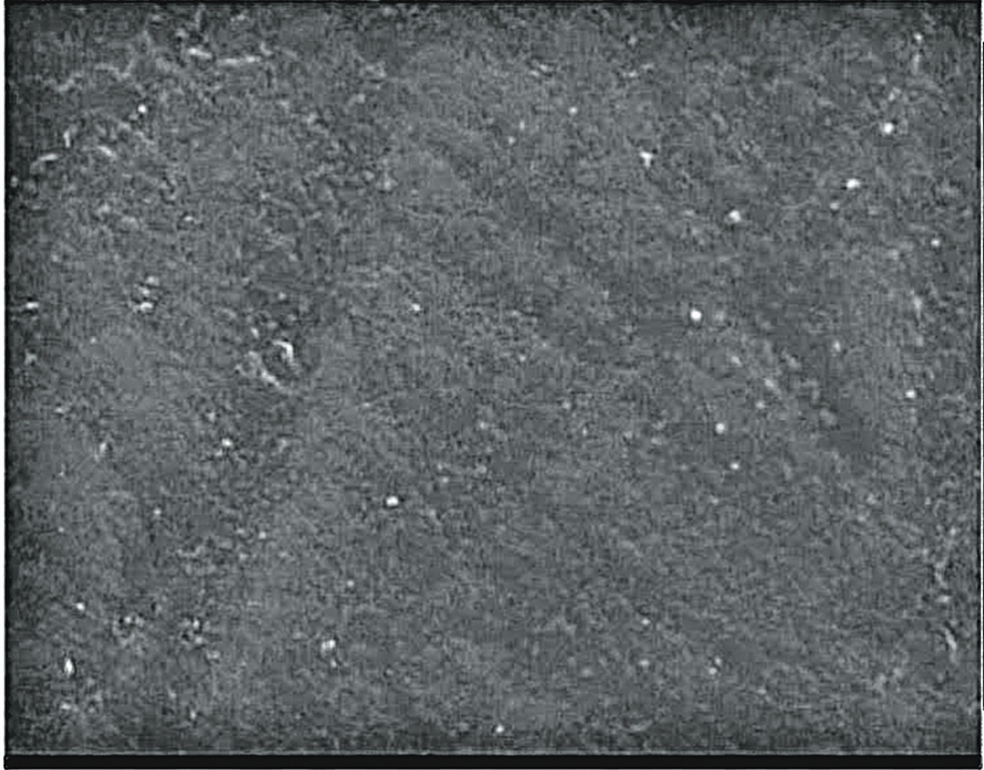
Demineralised enamel specimen.

The mean calcium and phosphorus values for healthy enamel samples were 65.25 ± 0.59 and 20.76 ± 0.81, respectively. Following demineralization, the mean values for calcium and phosphorus were 55.030.59 and 15.590.48, respectively. Following remineralization with strontium-doped nHAp, the calcium and phosphorus levels were 65.4 ± 0.86 and 20.5 ± 0.76, respectively. The mean values of calcium and phosphorus after remineralization using strontium-doped nanohydroxyapatite were higher and statistically significant (p < 0.05) when compared to the mean values of the demineralized specimen (Table 1, Figure 3). The average calcium and phosphorus content of each demineralized sample was determined using EDAX and SEM (Figures 3, 5, Table 1).

**Figure 6:**
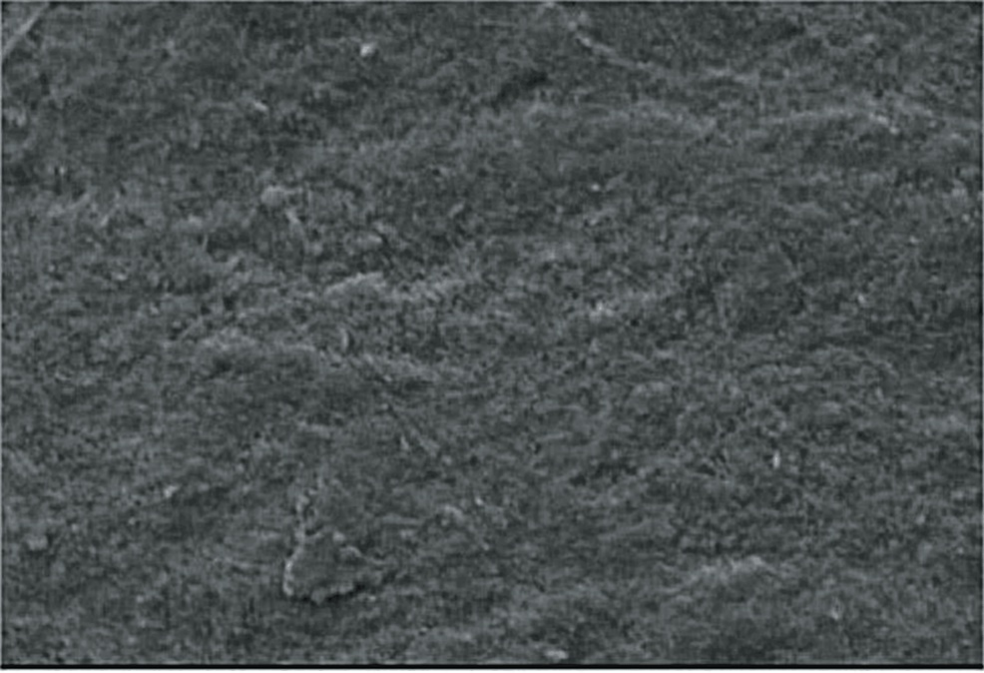
Remineralized enamel specimen.

Following remineralization, the samples were analyzed using SEM-EDAX examination to determine the mean amount of calcium and phosphorus content (Figures 3, 6, Table 1). After remineralization, the values were practically identical to the calcium and phosphorus values of sound enamel specimens.

SEM smooth surfaces may be seen in the images of the sound enamel specimens. Specimens after demineralization had a rough, irregular surface with pores. After remineralization, the specimens displayed smoother surfaces with greater mineral deposition when treated with the strontium-doped nHAp paste.

## Discussion

The early lesions and the white patches induce microscopic damage to the enamel, which results in cavities. It is possible that such cavities do not require a standard invasive restorative surgery. Non-cavitated lesions and decay extending to the dentin-enamel junction can be halted if specific microenvironmental pathogenic challenges are properly controlled or/and if therapeutic medications are applied for tissue healing [14].

Remineralizers are currently being applied to early lesions in an effort to curb demineralization and encourage remineralization. Many remineralizers have been developed, including fluoride, calcium supplements, and hydroxyapatite itself. nHAp has several limitations, including large crystal size, unsure biocompatibility, a lack of strength, brittleness, a high degree of crystallinity, and low solubility at neutral pH, requiring an acidic pH for dissolution [10]. Additionally, the endurance of nHAp is insufficient to maintain the enamel surface, leaving it vulnerable to plaque buildup (caused by the rough surface) and acid attacks from oral bacteria [12]. To resolve these problems, Krishnan et al. used various nHAp concentrations in their investigation. According to reports, strontium (Sr) and calcium (Ca) combine to form hydroxyapatite (HA), a pure, non-pressurized hydroxyapatite with a low (Sr + Ca)/P ratio. This partial replacement of Ca^2+^ ions by Sr^2+^ ions in the HAp substrate is what gives HAp its heightened acidic reactivity and insolubility. Sr-Hap with a greater than 10 mol% substitution has been discovered to be more soluble, and the increased bioactivity owing to the release of Sr^2+^ makes it more desirable in vivo [15].

In the study by Krishnan et al., 25% Sr-nHAp was the ideal material for enamel repair/regeneration because rising crystallinity and decreasing particle size improve diffusion through small lesions and areas with soft white patches [12]. However, Sr-doped hydroxyapatite was employed in the study in solution form, making it challenging to apply to tooth surfaces. It will be quite challenging to keep the liquid form on the tooth surface. Consequently, a novel paste including strontium-doped nHAp was developed in our study. The paste easily adheres to the tooth surface and makes this formulation just as simple to use as traditional toothpaste. A toothpaste composition investigation was conducted. EDAX can be used to ensure that the strontium, calcium, and phosphorus doping necessary for mineralization are integrated. For strontium-doped nHAp (EDAX) pastes, an elemental analysis found 21.99% Sr, 25.44% calcium, and 15.74% phosphorus. The EDAX peak strontium levels serve as confirmation of the impact of substituting strontium for calcium in nHAp. The SEM image of the paste sample revealed spherical particles. The effectiveness of Sr-nHAp has been demonstrated, and its potential for remineralization can be linked to its smaller particle size, which aids in pore penetration, and smaller crystal size, which boosts solubility. In this study, it was also discovered that strontium-doped nHAp in solution was superior to ACP-CPP cream and nHAp toothpaste in surface restoration of enamel demineralization [12]. According to the study, strontium was a superior option to CPP-ACP and Novamin for remineralizing or repairing enamel due to its higher material solubility and improved retention on the tooth surface.

Enamel samples were put in McInnes solution to mimic caries demineralization. The microhardness of the enamel was considerably decreased by demineralization using McInne bleaching solution [16]. To replicate the oral environment, fake saliva was used to preserve all samples. With the aid of EDAX, phosphorus values were calculated. For assessing mineral gain or loss in experimentally produced early caries lesions, EDAX is regarded as the gold standard. Mineral content may be measured quantitatively with extreme precision using EDAX [17,18]. At the superstructure level, elemental analysis is performed using EDAX. It is a microanalysis method that works in tandem with SEM, with EDAX handling the elemental analysis while SEM handles the structural analysis [19]. SEM was used to analyze the glaze topography changes in healthy, demineralized, and remineralized enamel samples. The topography and elemental enamel alterations may be clearly seen in the SEM-EDAX examination of the sample. The calcium and phosphorus readings were practically the same as those of natural teeth following remineralization with a new toothpaste. In comparison to the demineralized samples, there was a statistically significant rise in the mean calcium and phosphorus levels of the remineralized samples. Comparing the surface pores of the demineralized tooth samples, which display an open porous structure, made it clear that the surface pores were blurred.

When compared to a conventional dentifrice, the new strontium-doped nHAp paste showed reduced cytotoxicity and better cell survival in a second investigation to assess its cytotoxic effects [13].

This innovative strontium-doped nHAp paste was an effective treatment for early-stage carious lesions and white spot lesions due to its enhanced remineralization capability. Patients who received orthodontic treatment and developed white spot lesions after having their brackets removed may find it helpful. A study by Enezei et al. reported that strontium added with epidermal growth factor produced bone regeneration which replicated preosteoblasts, providing promising results [20].

Hence, different concentrations of strontium-doped nHAp in the paste form to ensure maximum remineralization may be undertaken in the future. Strontium-doped nHAp paste can be helpful in regular dental practice as it is a non-fluoride-based remineralizing agent. In vivo tests may also be performed to evaluate its remineralization potential and a comparison with existing remineralizing agents available in the market may be done before clinical application. Further clinical trials are required to prove the in vitro results obtained.

## Conclusions

The novel strontium-doped hydroxyapatite paste showed good remineralization potential. SEM-EDAX evaluation showed favorable topographic changes in enamel. Further comparison of the remineralization potential of strontium-doped hydroxyapatite paste with other remineralization agents and evaluation of its physical characteristics will provide the basis for support of the outcome of this study. However, further evaluation with different concentrations of strontium-doped nHAp in the paste form to ensure maximum remineralization needs to be undertaken.
